# Synergistic effects of foliar potassium supplementation with photoperiod on storage tolerance and taste of fresh cut lettuce

**DOI:** 10.3389/fpls.2025.1627792

**Published:** 2025-08-07

**Authors:** Wenyuan Wang, Muhammad Mahmood Ur Rehman, Jizhan Liu, Shuo Wu, Shengyi Zhao

**Affiliations:** ^1^ School of Agricultural Engineering, Jiangsu University, Zhenjiang, China; ^2^ Key Laboratory of Modern Agricultural Equipment and Technology, Ministry of Education, Jiangsu University, Zhenjiang, China; ^3^ National Digital Agriculture Equipment (AI & Agribot) Innovation Sub-center, Jiangsu University, Zhenjiang, China

**Keywords:** environmental effect, growth, storage, taste, antioxidant

## Abstract

Fresh cut lettuce (*Lactuca sativa L.*) is an important leafy vegetable, due to high perishability, storage tolerance become a critical factor to maintain its quality and taste. This study aimed to determine whether photoperiod duration and potassium fertilizer will boost the taste and the storage tolerance of fresh cut lettuce. A factorial experiment comprising two factors was conducted, involving two photoperiod durations (14h light/day and 12h light/day) and various concentrations of potassium fertilizer (0%, 0.3%, 0.6% and 0.9%) as foliar sprays. For fresh cut lettuce cultivated under a 14-hour photoperiod, applying either 0% or 0.3% potassium enhanced water content, soluble solids accumulation, supported the maintenance of antioxidant enzyme activity stability, and reduced the decay rate during storage. While 0.3% or 0.6% potassium concentration was required to achieve the high quality and storability aim under 12h photoperiod. Current findings may provide a valuable information toward sustainable strategies for enhancing the post-harvest life of fresh cut lettuce with maintained taste and its potential application in post-harvest management of commercial produce.

## 1 Introduction

Fresh cut lettuce (*Lactuca sativa L.*) is a processed product derived from fresh vegetables. With the growing demand for convenient food options, the market demand for fresh cut vegetables in China is also on the rise. However, current research on fresh cut vegetables mainly focuses on the screening of varieties, processing techniques, preservation technologies, evaluation models and physiological changes (Alenyorege et al., 2019; Zhang et al., 2021; Chen et al., 2025; Ji et al., 2025; He et al., 2025). Alongside the storage environment and technologies, to some extent, the shelf life of fresh cut vegetables also influenced by their own morphological and physiological characteristics. Moreover, in addition to the varietal traits, the environmental conditions experienced during cultivation significantly influence these physiological characteristics. Consequently, the shelf life of fresh cut vegetables is largely determined by the environmental factors present during their growth stages.

Potassium, an essential nutrient for crop growth and development, is often referred to as the “quality element” by researchers with its versatile nature of application for crop improvement (Wang and Wu, 2009; Mahmood ur Rehman et al., 2024). Applying the right amount of potassium fertilizer can improve fruit quality and prolong their storage life at high quality. For example, Tavallali et al. (2018) found that increasing potassium fertilizer supply boosts phenolic content in tomato fruit and enhance their antioxidant capacity during storage. The influence of potassium on fresh cut vegetables is controversial, since both positive and negatives effects have been observed. Hoque et al. (2010) showed that the application of the potassium fertilizer did not affect the post-harvest quality of stored lettuce, while Chrysargyris et al. (2018) show that potassium deficiency will reduce the antioxidant capacity of hydroponic fresh cut lettuce. Besides, due to the antagonistic interaction between potassium and either calcium and magnesium ions, excessive potassium can reduce calcium and magnesium levels in fruits, leading to decreased firmness at harvest and tissue collapse during fruit storage (Alva et al., 2006; Okba et al., 2021). Therefore, only the reasonable application of potassium fertilizer can effectively improve the taste and shelf life of fresh cut fruits and vegetables.

Light serves as the fundamental driver for photosynthesis in plants, with light quality, intensity, and photoperiod each exerting distinct influences on the quality and storability of fruits and vegetables. For instance, within a certain range, extending the photoperiod can effectively mitigate the yellowing, softening and decay of fruit and vegetable tissues, however, excessive illumination may yield adverse effects (Lai et al., 2022; Ziaf et al., 2017; Guo et al., 2023; Brito et al., 2023). The mechanism through which light influences plant oxidative stress is multifaceted. It is widely accepted that light enhances anti-aging effects by stimulating the production of antioxidant components, such as phenolic compounds and ascorbic acid (Charles et al., 2018; Zhan et al., 2012). Additionally, certain light sources have been shown to extend storage life by inactivating pathogens and exerting antibacterial effects (Zhu, 2022). Nevertheless, in practical agricultural production, the quality of fruits and vegetables is shaped by a combination of factors. Briefly, the duration, intensity and quality of light affect the potassium nutrition regulation and eventually crop quality (Ahammed et al., 2022).

To date, few studies have explored how the interaction between photoperiod and potassium concentration during cultivation can synergistically enhance the quality and storability of fresh cut lettuce. Therefore, this study aims to identify optimal photoperiod - potassium concentration combinations via integrated LED lighting and foliar fertilization management to maximize fresh cut lettuce quality and shelf life, providing practical guidance for cultivation practices.

## 2 Materials and methods

### 2.1 Cultivation of romaine lettuce

This study was performed at Jiangsu University (School of Agricultural Engineering) between November 2023 and February 2024. Seeds of romaine lettuce (*Lactuca sativa L.* var. Italy 338), purchased from Shouguang Xinxinran Horticulture Co., Ltd, Shandong, China and grown in the laboratory. Seedlings were transplanted in potts (14×14×25cm) at a spacing of 20cm×20cm in intelligent artificial climate chamber (Ningbo Safe Experimental Instrument Co., Ltd, Zhejiang, China, PRX-600C) as they reached three leaves stage with one heart. The day/night temperature was 25 ± 1°C/18 ± 1°C, the relative humidity (RH) was 60 ± 5%, and the CO2 concentration was ambient. The commercial substrate (purchased from Hebei Dewoduo Biotechnology Co., Ltd, Hebei, China) consists of sphagnum peat, coconut coir dust and perlite, supplemented with controlled-release fertilizer. The nutrient solution was applied every 7 days, using the Hoagland formula (Li and Cheng, 2015). The pots were arranged in randomized block design with three replicates for each treatment, while nine plants were used for each replicates.

### 2.2 Experimental design

The factorial experiment was designed focusing on photoperiod and foliar potassium application. The light source was the built-in LED system of the intelligent artificial climate chamber, with a photosynthetically active radiation (PAR) of 80 ± 5μmol m^−2^ s^−1^ measured by Tuopu Yunong quantum meter. During preliminary trials, three photoperiod (10h, 12h and 14h light/day) were tested. Observations revealed that 10h-light duration resulted in undesirable growth characteristics for the lettuce variety, including yellowish leaves, narrow and elongated leaf morphology, and extended internode spacing. Consequently, the formal experiment was conducted with two photoperiod duration: 14h light/day and 12h light/day. For potassium application, potassium chloride was applied at four concentrations: 0%, 0.3%, 0.6% and 0.9%. The spraying process was carefully controlled to ensure complete leaf coverage without dripping, and applications were repeated every five days. Treatment began on the 10^th^ day after transplanting. The harvest time was on the 40^th^ day after transplanting, and the harvest criteria were full plant shape, no pests or diseases, and no obvious mechanical damage.

The test samples were collected in the early morning, lettuce roots were removed with a sterilized stainless steel knife, and then clean leaf with uniform size was selected in the middle of lettuce. Leaves were packaged in plastic bags, with 10 leaves in each bag, and were repeated five times. Samples for measuring quality and enzyme activity-related indicators were stored in an ultra-low temperature refrigerator, while samples for measuring moisture content and decay rate-related indicators were stored in a conventional refrigerator at 10°C.

### 2.3 Measurement indicators and methods

#### 2.3.1 Taste indicators

Some of the taste indicators including hardness, springiness, chewiness, gumminess, resilience, cohesiveness, water content and soluble solids content were measured. For this purpose, samples were collected on the day of harvest (marked as Day 0 of storage) and on Day6 of storage for measurement (marked as Day 6).

The measurements of hardness, springiness, chewiness, gumminess, resilience, cohesiveness were taken using a texture analyzer (SMS TA) equipped with a cylindrical probe P/36mmR with a diameter of 36 mm. The parameter settings were based on the methods described by Liu (2013) and Hu et al. (2022), with trigger value of 5g, test rate of 2 mm/s, shear level of 1cm at the bottom of the plate, cycle count of 2, and maximum force set to the default value in the software. Six samples were tested each time, and the average value was taken.

The water content were determined using the oven drying method. By weighing a fresh sample and record it as A and then placed it in an oven at 105°C and dried it until it reached to a constant weight. After cooling, the dry weight of the sample was record as B. The moisture content of the sample is represented by (A-B).

The soluble solids were measured using a handheld sugar refractometer. For each treatment, three freshly cut lettuce leaves were taken, ground into a homogenate using a mortar, and 2 drops were aspirated for measurement.

#### 2.3.2 Storage tolerance index

The determination indicators including decay rate, malondialdehyde (MDA), superoxide dismutase (SOD), peroxidase (POD), and catalase (CAT) were measured. Sampling was initiated on Day 0 and taken every 2 days for a total of 4 sampling time (Day 0, Day 2, Day 4 and Day 6). Lettuce leaves without visible damage or disease were selected for analysis. Samples were immediately stored in an ultra-low temperature freezer (-80°C) after collection. MDA and enzyme activities (SOD, POD, CAT) were measured via Elisa kits (purchased from Jiangsu Meimian Industrial Co., Ltd) according to the manufacturer’s instructions.

Formula for calculating decay rate:


Decay rate (%)=Number of rotten lettuce on Day 6 / Total number × 100%

### 2.4 Data analysis

Data statistical analysis was conducted using SPSS (21.0) for ANOVA, and Duncan’s multiple comparison test was employed for significance analysis. A P-value< 0.05 indicated significant differences at the 0.05 level. Data visualization was performed using Origin Pro 2023 software.

## 3 Results and discussion

### 3.1 The impact of light and potassium application on the taste of fresh cut lettuce

#### 3.1.1 Water content

**Figure 1:**
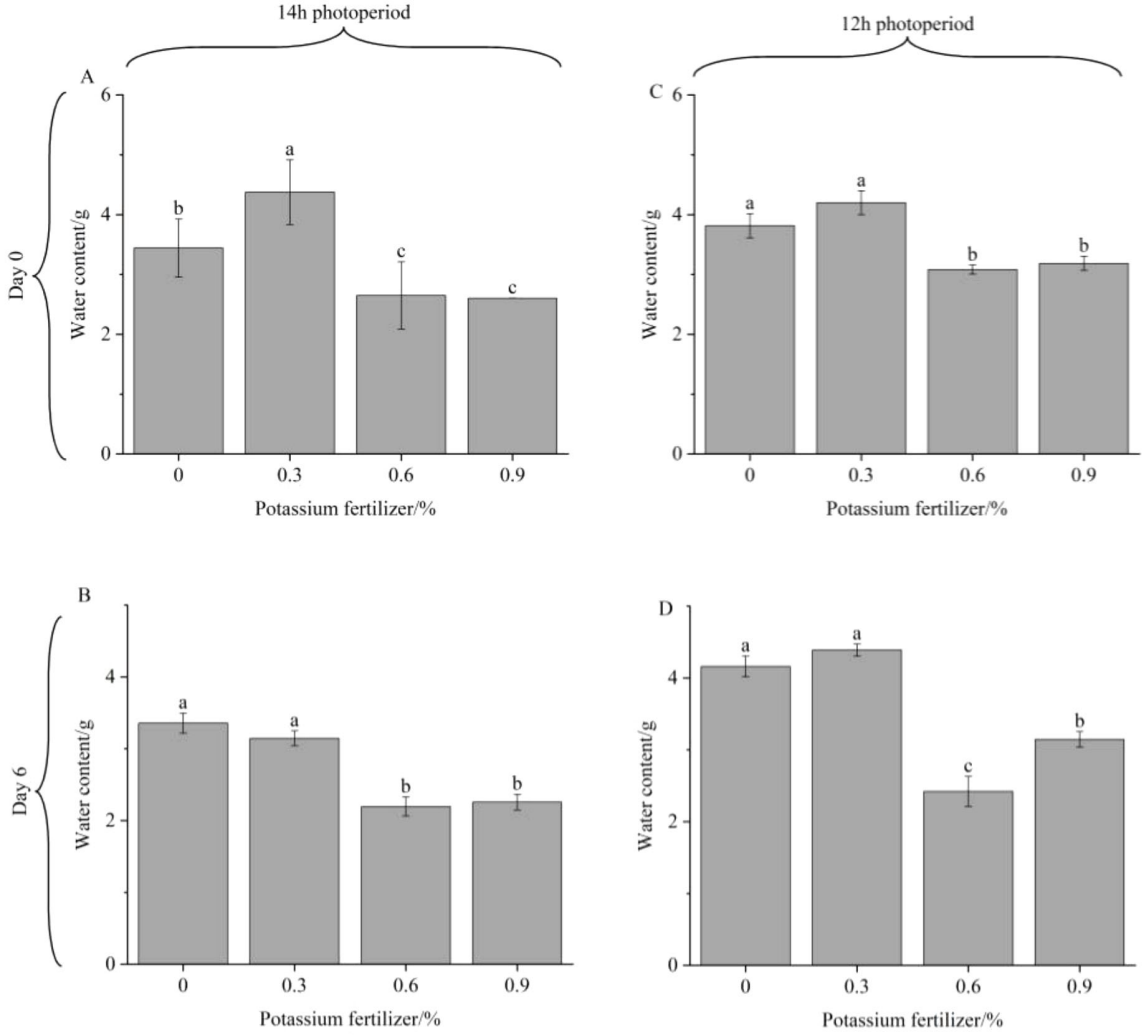
Water content of fresh cut lettuce cultured at 14h photoperiod **(A, B)** or 12h photoperiod **(C, D)** with potassium fertilize of 0%, 0.3%, 0.6% and 0.9%, respectively. Statistical significances (p<0.05) of values in the same storage time are indicated by different letters.

During storage, vegetables undergo a series of complex physiological activities, including water evaporation and nutrient metabolism, which result in a continuous decline in quality (Shen et al., 2022). It is widely recognized that higher water content in plants correlates with greater vitality and more active metabolic activity (Zhu et al., 2023; Zhang et al., 2024). As show in **Figure 1**, the difference of water content between Day 6 and Day 0 is small, which may be attributed to two primary factors: first, the use of plastic bags in the experiment effectively minimized water evaporation and loss. Second, the small temperature change between the storage environment and the growth temperature, along with the consistent storage temperature, likely reduced the respiratory intensity and ethylene production of the post-harvest fresh cut lettuce, this is similar to the experimental conclusion of Gorny et al. (2000); Martín et al. (2025).

Throughout the storage time, regardless of the photoperiod, lettuce grown at 0% potassium fertilizer and 0.3% potassium fertilizer always had a significantly higher water content than lettuce treated with 0.6% potassium fertilizer and 0.9% potassium fertilizer. These results demonstrate that in this experiment, compared to the 0.6% or 0.9% potassium fertilizer, 0% or 0.3% potassium fertilize was more conducive to water absorption and accumulation in fresh cut lettuce, consequently producing more tender harvested lettuce. Fang et al. (2023) demonstrated that elevated foliar potassium concentrations enhance leaf water content by reducing osmotic potential, thereby facilitating water uptake. However, due to the antagonistic interaction between potassium and calcium, excessive potassium can reduce calcium levels (Alva et al., 2006; Okba et al., 2021), which is crucial for activating the phosphorylation of aquaporin kinases, playing a role in regulating aquaporin channel activity. Insufficient calcium leads to narrowed aquaporin channels and reduced water permeability (Balasubramanian et al., 2008). Our study confirmed this causal relationship: optimal potassium concentrations enhance water content in fresh cut lettuce, and the positive effect was only observed within an optimal potassium concentration range, beyond which osmotic stress occurred.

#### 3.1.2 Texture parameters

The Texture Profile Analysis (TPA) test conducted by the texture analyzer is designed to mimic human chewing motions. By employing mechanical approaches, it accurately measures the texture properties of the sample under examination. This test is capable of providing a comprehensive reflection of the texture characteristics of the tested object, thereby holding significant fundamental importance for elucidating the texture features of fruits and vegetables (Jiang et al., 2023; Qianqian et al., 2024).

**Table 1** show the texture characteristic parameters of fresh cut lettuce treated at Day 0. As shown in **Table 1**, the photoperiod-fertilizer coupling treatment in this experiment demonstrated significant effects on the hardness, chewiness, and gumminess of fresh cut lettuce (P<0.05), while showing no significant influence on springiness, resilience, and cohesiveness. Gumminess describe the energy required to decompose sample before swallowing. Chewiness refers to the amount of energy required to grind a solid product to a swallowable state, the lower chewiness, the easier the product will be swallow (Tian et al., 2024). So the combination of 14h-0.9% and 12h-0.3% were particularly effective in maintaining the quality of fresh cut lettuce and developing the fundamental palatability characteristics producing firmer texture and better chewiness while maintaining appropriate gumminess levels.

**Table 1:** Texture characteristic parameters at Day 0 of fresh cut lettuce cultured at 14h photoperiod or 12h photoperiod with potassium fertilize of 0%, 0.3%, 0.6% and 0.9%, respectively.

Index	Potassium fertilize/%	14h photoperiod	12h photoperiod
Hardness/g	0	147.75 ± 45.86 b	269.52 ± 116.41 b
	0.3	153.44 ± 86.31b	369.84 ± 58.14 a
	0.6	182.30 ± 53.53 ab	254.14 ± 13.22 b
	0.9	352.50 ± 144.23 a	294.33 ± 172.25 b
Springiness/mm	0	0.94 ± 0.03 a	0.93 ± 0.04 a
	0.3	0.91 ± 0.10 a	0.86 ± 0.06 a
	0.6	0.93 ± 0.23 a	0.75 ± 0.01 a
	0.9	0.89 ± 0.09 a	0.91 ± 0.06 a
Chewiness/mJ	0	5.49 ± 0.37 b	10.40 ± 0.05 b
	0.3	5.11 ± 0.10 b	24.14 ± 2.92 a
	0.6	5.96 ± 2.15 b	10.13 ± 0.02 b
	0.9	27.29 ± 5.95 a	13.04 ± 3.95 ab
Gumminess/g	0	4.28 ± 1.96 a	2.54 ± 1.17 b
	0.3	1.16 ± 0.10 b	2.98 ± 0.49 a
	0.6	0.85 ± 0.43 c	1.89 ± 0.32 ab
	0.9	3.76 ± 0.24 ab	2.30 ± 1.154 ab
Resilience/mm	0	0.57 ± 0.15 a	0.56 ± 0.18 a
	0.3	0.67 ± 0.13 a	0.49 ± 0.06 a
	0.6	0.56 ± 0.25 a	0.44 ± 0.10 a
	0.9	0.57 ± 0.04 a	0.51 ± 0.20 a
Cohesiveness	0	0.79 ± 0.05 a	0.93 ± 0.05 a
	0.3	0.69 ± 0.27 a	0.81 ± 0.04 a
	0.6	0.77 ± 0.13 a	0.69 ± 0.12 a
	0.9	0.76 ± 0.04 a	0.80 ± 0.16 a

Statistical significances (p<0.05) of values in the same storage time are indicated by different letters.

The TPA test results at Day 6 showed that all indicators were significantly lower than those measured at Day 0, and there were no significant differences between treatments, so that results were not presented here. This could be attributed to the destruction of superficial layer cells and the degradation of internal structures (Zhang et al., 2019; Mustapha et al., 2019; Martín et al., 2025). After cutting, the tissue structure is destroyed and the juice is outflowed, the regionalization of enzymes and substrates in lettuce is destroyed, triggering a series of physiological and biochemical reaction, including cell wall decomposition and cell membrane destruction (Qianqian et al., 2024).

#### 3.1.3 Soluble solids content

**Figure 2:**
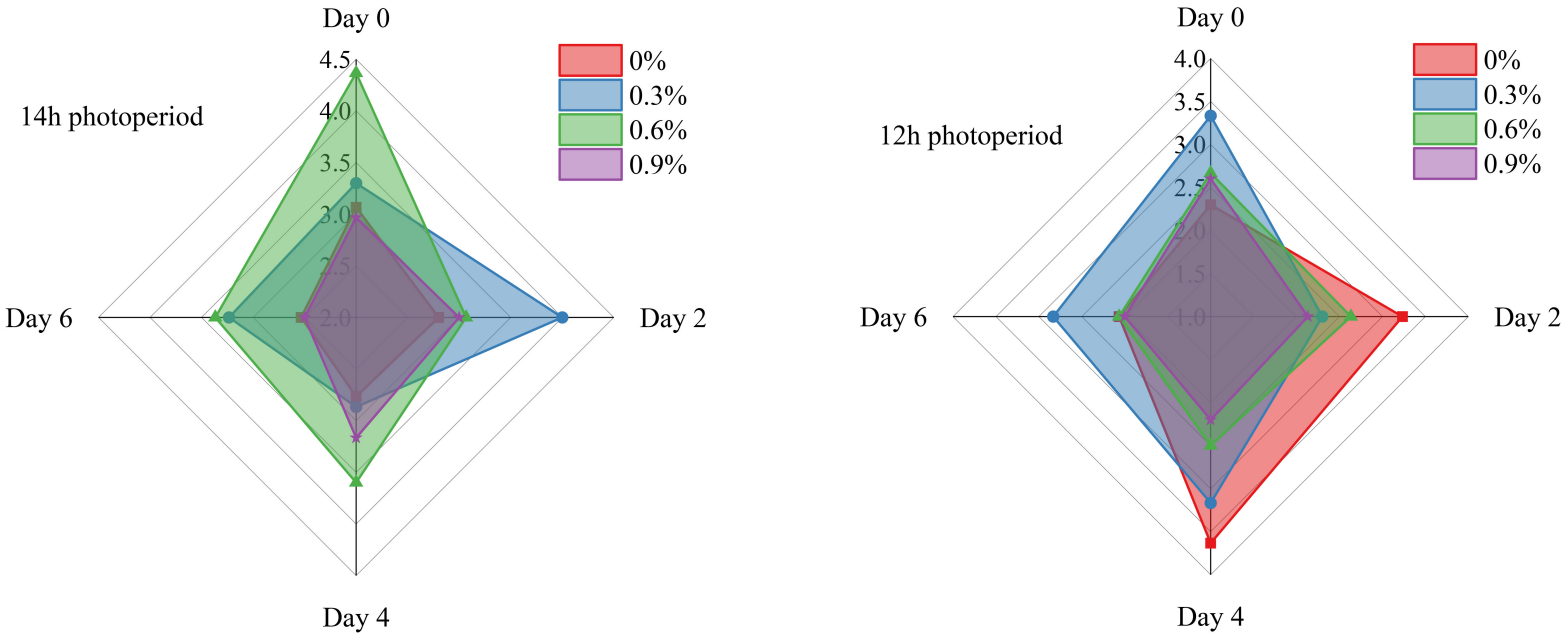
Soluble solids content of fresh cut lettuce cultured at 14h photoperiod or 12h photoperiod with potassium fertilize of 0%, 0.3%, 0.6% and 0.9%, respectively. The numerical values on the vertical axis of the radar chart represents the soluble solids content (%). Specifically, the maximum scale for soluble solids content is 4.5% in the 14h photoperiod radar chart and 4% in the 12h photoperiod radar chart.

**Figure 2** illustrates the changes in soluble solids content of fresh cut lettuce during the whole storage time. We can clearly observe from the figure that on Day 0, under a 14-hour photoperiod, 0.6% potassium concentration is more conducive to the accumulation and stabilization of soluble solids in fresh cut lettuce, whereas under a 12-hour photoperiod, the optimal concentrations are 0% or 0.3%. Maybe it related to factors such as photosynthesis and enzyme activity. The longer 14h photoperiod provides fresh cut lettuce with more ample lighting time, facilitating the continuous progress of photosynthesis. Meanwhile, a potassium concentration of 0.6% can optimize the activity of photosynthesis-related enzymes, creating a better environment for the conversion and transport of photosynthetic product, enabling more of them to transported and accumulated (Cui and Tcherkez, 2021). In contrast, the 12h photoperiod is relatively short. Under such circumstances, an excessively high potassium concentration (such as 0.6%) may disrupt the ion balance within the plant, thereby affecting the transportation and accumulation of photosynthetic products.

As a whole, the soluble solids content of fresh cut lettuce in all treatments on Day 6 was significantly lower than that on Day 0. This is attributed to the accumulation of starch during the growth phase of fruits and vegetables, which provides support to the cells. With the maturity of fruits and vegetables, starch is continuously hydrolyzed into soluble sugars, leading to a gradual increase in soluble solids content and a decrease in cell tension. As fruits and vegetables continue to mature, soluble solids become respiratory substrates to be consumed, thus resulting in a decreasing content (Li et al., 2024). So, in numerous studies, researchers consider the time when the soluble solids in fruits and vegetables reach their peak as a crucial indicator for assessing their ripening and senescence. The earlier the soluble solids peak, the faster the quality declines and the poorer the storability (El-Mesery et al., 2024; Li et al., 2011). In this experiment, when fresh cut lettuce was grown under a 14h photoperiod, the treatment with 0.3% potassium concentration exhibited the latest occurrence of soluble solids peak (Day 2), while the soluble solids peak of other treatments appeared on Day 0. Similarly, when fresh cut lettuce was grown under a 12h photoperiod, the treatment with 0% potassium concentration demonstrated the latest peak of soluble solids, which occurred on Day 4.

### 3.2 Impact of various treatments on the storage tolerance of fresh cut lettuce

#### 3.2.1 Decay rate

**Figure 3:**
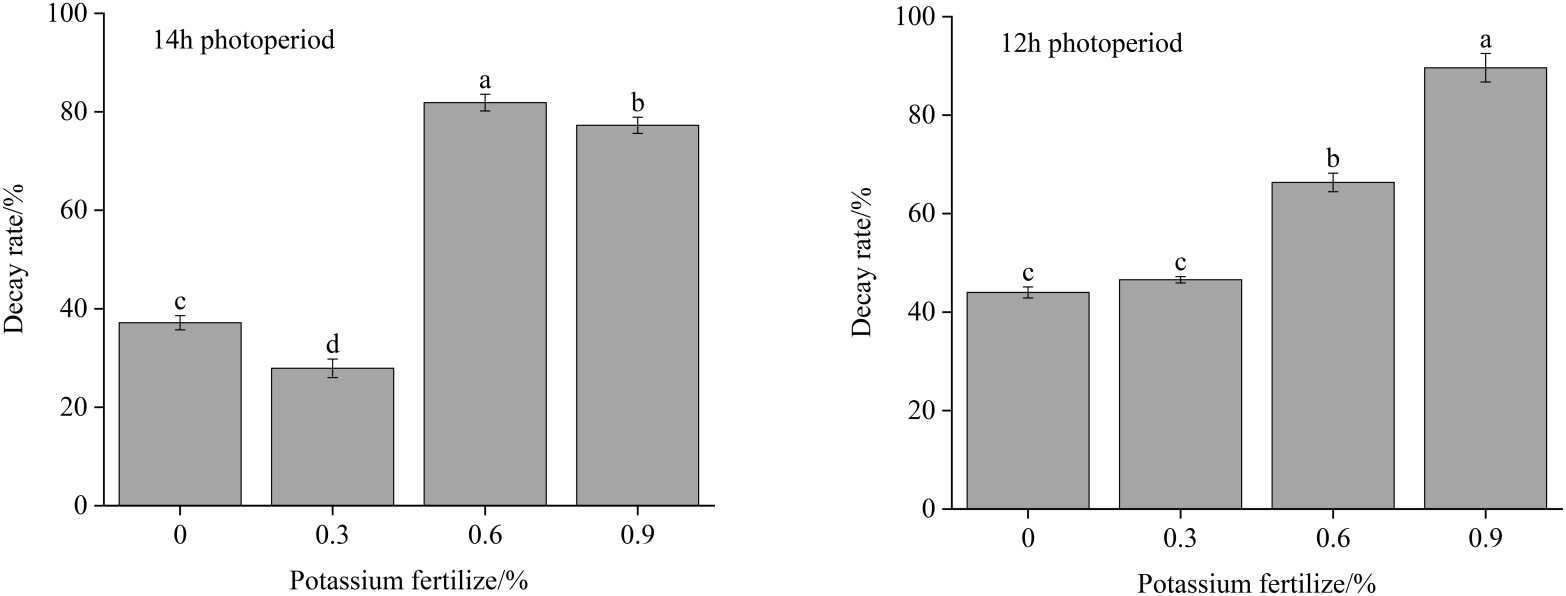
Decay rate of fresh cut lettuce cultured at 14h photoperiod or 12h photoperiod with potassium fertilize of 0%, 0.3%, 0.6% and 0.9%, respectively. Statistical significances (p<0.05) of values in the same storage time are indicated by different letters.

**Figure 3** shows the decay rate of fresh cut lettuce under various treatments on Day 6. As shown in **Figure 3**, regardless of the photoperiod, lettuce grown at 0.6% potassium fertilizer or 0.9% potassium fertilizer always had a significantly higher decay rate than lettuce grown at 0% potassium fertilizer or 0.3% potassium fertilizer. That may due to the fact that an appropriate concentration of potassium can maintain the osmotic balance between the inside and outside of cells, ensuring the normal turgor pressure of cells (Ahmad et al., 2023). This helps to reduce tissue softening during storage, delay senescence and reduce the decay rate. Additionally, when potassium is present at optimal concentrations, it can decrease plants’ susceptibility to disease (Elad et al., 2021). A similar conclusion was reached with fresh cut lettuce in this experiment. However, it is imperative to discern whether this effect is attributable to the chloridion ion present in KCl applied in this experiment, because according to the research by Yang et al. (2024), pre-irrigation with chloride salt improved plant defense against the fungal pathogen challenge.

**Table 2** displays the significance test results evaluating the effects of photoperiod, foliar potassium fertilizer, and their interactions on the decay rate of fresh cut lettuce. Both individual factors and their combined interactions significantly influence the decay rate of fresh cut lettuce, especially foliar potassium fertilizer. But it’s clear that the effect of foliar potassium fertilization on the decay rate is not a straightforward linear relationship, overall, beyond the appropriate concentration range, potassium foliar spraying is not beneficial to the storability of fresh cut lettuce.

**Table 2:** Significance test of decay rate of fresh cut lettuce in each treatments (F) (∗∗p < 0.01).

Index	Photoperiod	Fertilizer	Photoperiod×Fertilizer
Decay Rate	20.774**	129.741**	14.029**

#### 3.2.2 MDA

**Figure 4:**
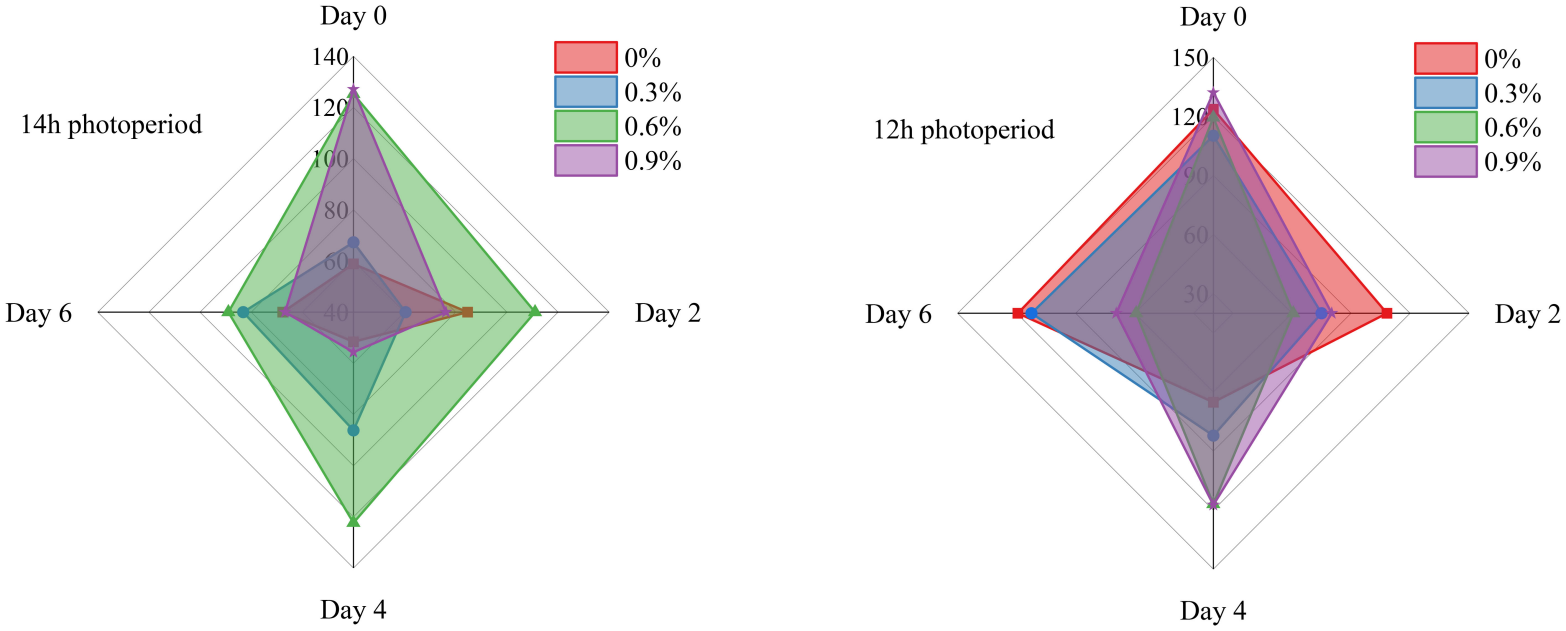
MDA of fresh cut lettuce cultured at 14h photoperiod or 12h photoperiod with potassium fertilize of 0%, 0.3%, 0.6% and 0.9%, respectively. The numerical values on the vertical axis of the radar chart represents the MDA (μmol/g). Specifically, the maximum scale for MDA is 140 μmol/g in the 14h photoperiod radar chart and 150 μmol/g in the 12h photoperiod radar chart.

**Figure 5:**
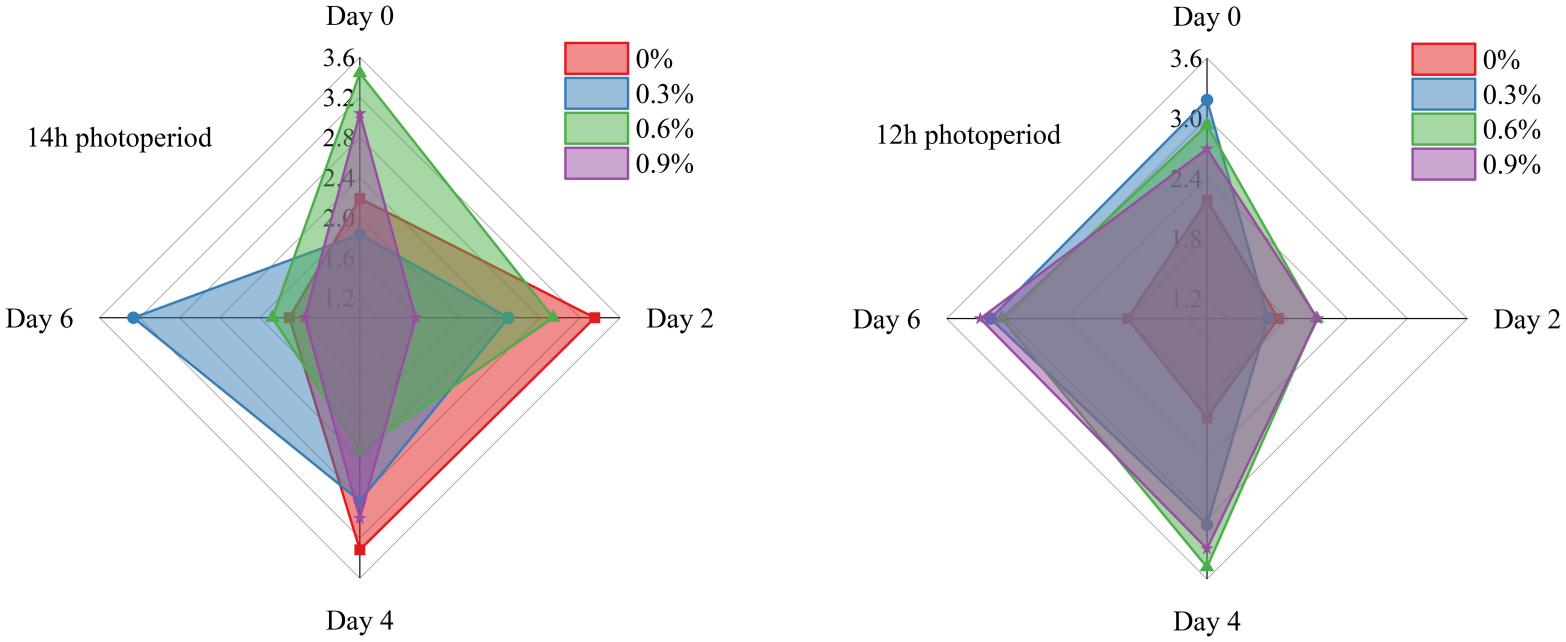
POD content of fresh cut lettuce cultured at 14h photoperiod or 12h photoperiod with potassium fertilize of 0%, 0.3%, 0.6% and 0.9%, respectively. The numerical values on the vertical axis of the radar chart represents the POD content (μ/g). The maximum scale for POD content is 3.6 μ/g in 14h photoperiod radar chart and 12h photoperiod radar chart.

**Figure 4** illustrates the changes in MDA in fresh cut lettuce in this experiment. MDA, which serves as the terminal product of lipid peroxidation, exhibits cytotoxic properties, and its concentration demonstrates a close correlation with the degree of membrane damage (Wang et al., 2017). In general, there is a positive relationship between the intensity of stress and the level of MDA, the more severe the stress an organism experiences, the higher the concentration of MDA will be (Min et al., 2022; Tang, 2023). As show in **Figure 5**, when lettuce grown under 14h photoperiod, the MDA during the whole storage time initially increases and then decreases with rising potassium concentrations, while it exhibits an opposite trend under 12h photoperiod, demonstrate that potassium, as a stress alleviator nutrient, at an appropriate concentration can effectively reduce membrane damage and enhance lettuce storage resistance, which is consistent with many research findings (Farahani et al., 2019; Fang et al., 2022).

#### 3.2.3 Antioxidant enzyme activity

A key factor driving plant senescence is the accumulation of reactive oxygen species (ROS), that can directly disrupt cellular metabolism leading to cell death (Ahmad et al., 2016). However, antioxidants such as SOD, POD, and CAT play a crucial role in neutralizing ROS, significantly reducing their detrimental effects on cell membranes and thereby delaying plant senescence (Yang et al., 2022).

**Figure 6:**
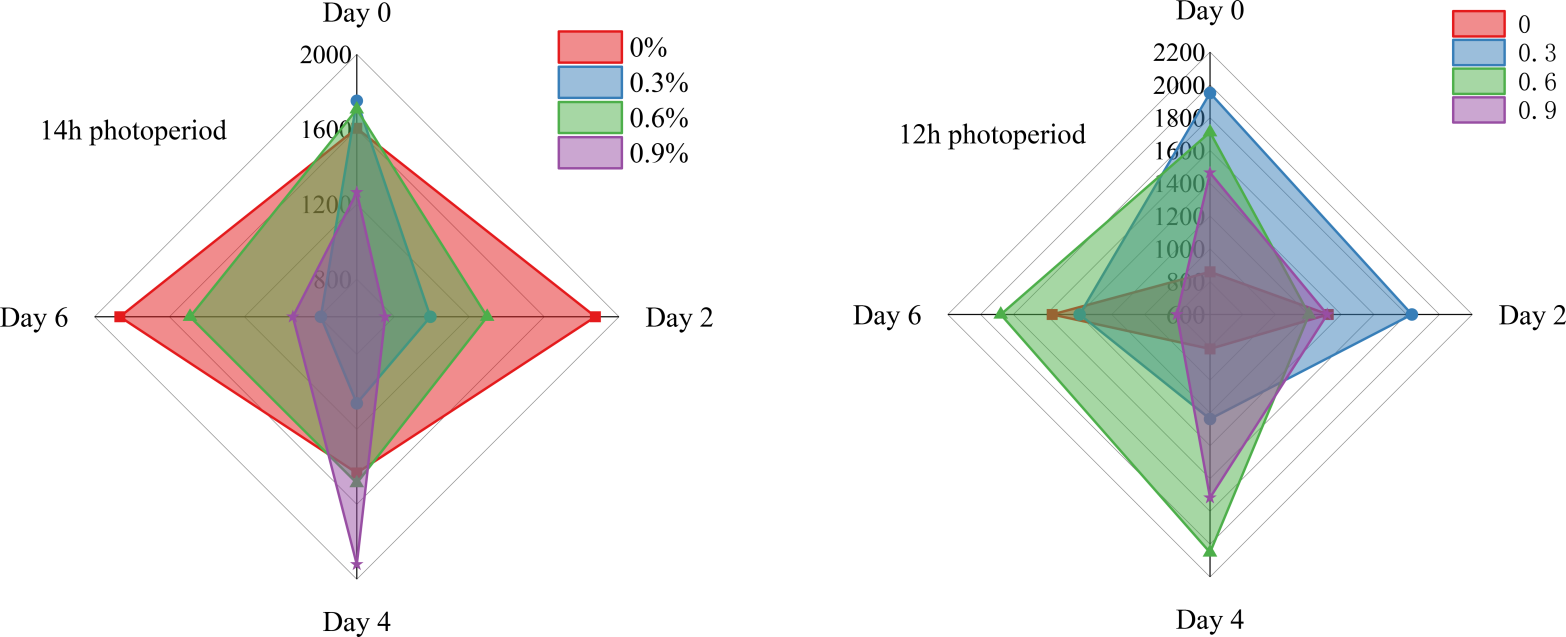
SOD content of fresh cut lettuce cultured at 14h photoperiod or 12h photoperiod with potassium fertilize of 0%, 0.3%, 0.6% and 0.9%, respectively. The numerical values on the vertical axis of the radar chart represents the SOD content (μ/g). Specifically, the maximum scale for SOD content is 2000 μ/g in the 14h photoperiod radar chart and 2200 μ/g in the 12h photoperiod radar chart.

**Figure 7:**
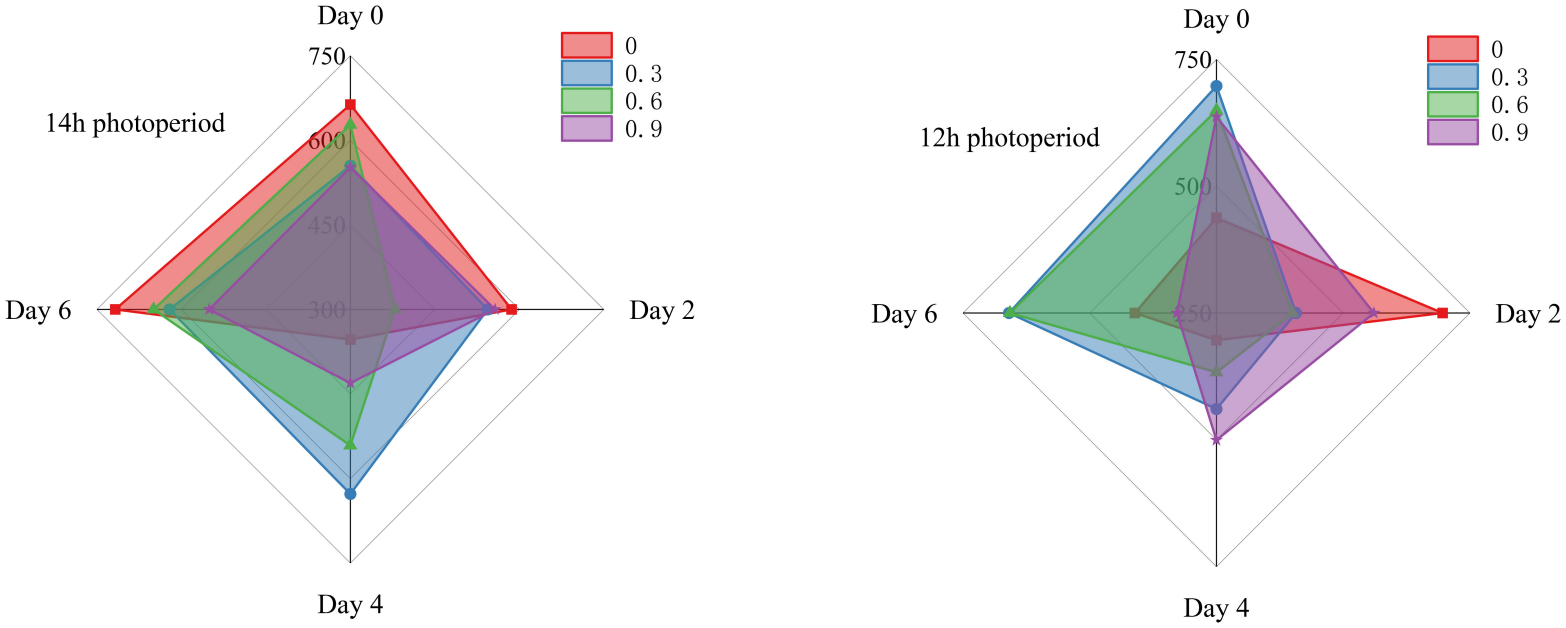
CAT content of fresh cut lettuce cultured at 14h photoperiod or 12h photoperiod with potassium fertilize of 0%, 0.3%, 0.6% and 0.9%, respectively. The numerical values on the vertical axis of the radar chart represents the CAT content (μ/g). The maximum scale for CAT content is 750 μ/g in 14h photoperiod radar chart and 12h photoperiod radar chart.

As shown in **Figures 5**–**7**, throughout the whole treatment, when fresh cut lettuce grown under 12h photoperiod, 0.3% and 0.6% potassium was more beneficial to maintain the stability of antioxidant enzyme activity, especially over a long storage time. When fresh cut lettuce grows under a 14h photoperiod, the situation becomes much more complex, but overall, it seems that the low concentrations of potassium are more beneficial for long-term storage with high antioxidant enzymes. In recent decades, extensive research has revealed that potassium plays a dual role in plants. It is not merely a constituent of plant structure but also exerts a significant influence on the normal functioning of numerous metabolic processes and the maintenance of ROS homeostasis (Hasanuzzaman et al., 2018; Houmani et al., 2022). Building on this existing knowledge, our research has found that the increased activity of antioxidant enzymes in plants may be attributed to the action of an appropriate concentration of potassium. This aligns with the research of Siddiqui et al. (2012), which indicated that potassium can activate more than 50 enzymes, suggesting a potential link between potassium - mediated enzyme activation and the enhanced antioxidant enzyme activity in our experiments.

Xun et al. (2025) and Zhu et al. (2023) have identified a significant negative correlation between decay severity and soluble solids, so they thought that enhancing the intrinsic quality of fruits and vegetables can improve their storability and disease resistance. In the present experiment, it can also be found that there is a certain correlation between the water content, soluble solids, decay rate, MDA content and antioxidant enzyme activity of fresh cut lettuce. Thus, we may postulate that in this experiment, the application of photoperiod and appropriate concentration of potassium together can effectively improve the quality of fresh cut lettuce at harvest, and the high quality at harvest is conducive to improving the storability of fresh cut lettuce.

## 4 Conclusions

In this experiment, we found that additional application of appropriate concentration of potassium could help alleviate the adverse effect of short photoperiod on fresh cut lettuce. When fresh cut lettuce grown under a 14h photoperiod, spraying 0% or 0.3% potassium was beneficial to the accumulation of water content, soluble solids, as well as maintaining the stability of antioxidant enzyme activity and reducing the decay rate during storage. While 0.3% or 0.6% potassium concentration was required to achieve the high quality and storability aim under 12h photoperiod. However, these findings were obtained under laboratory conditions and require further testing and validation in actual production settings to fully realize the potential of photoperiod-fertilizer combination effects for enhancing the quality and storage resistance of fresh cut lettuce.

## Data Availability

The original contributions presented in the study are included in the article/Supplementary Material. Further inquiries can be directed to the corresponding author.
